# Ileal Pouch-Anal Anastomosis for Ulcerative Colitis: Predictors of Early and Late Complications

**DOI:** 10.7759/cureus.75086

**Published:** 2024-12-04

**Authors:** Yajnadatta Sarangi, Ashok Kumar, Somanath Malage, Nalinikanta Ghosh, Rahul Rahul, Ashish Singh, Supriya Sharma, Rajneesh K Singh, Anu Behari, Ashok Kumar

**Affiliations:** 1 Department of Surgical Gastroenterology, Sanjay Gandhi Postgraduate Institute of Medical Sciences (SGPGIMS), Lucknow, IND

**Keywords:** factors associated with complications, ileal pouch-anal anastomosis, postoperative early and late complications, predictors of complication, ulcerative colitis

## Abstract

Background

Restorative proctocolectomy with ileal pouch-anal anastomosis (IPAA) is often considered the preferred surgical treatment for ulcerative colitis. This study was conducted to investigate the early and late complications of ileal pouch-anal anastomosis in patients with ulcerative colitis, as well as the factors associated with these complications.

Methodology

All relevant clinical and operative data of patients (n = 101) who underwent IPAA for ulcerative colitis between January 1995 and December 2018 were retrieved from a prospectively maintained database. Early complications, various late complications, and their predictive factors were studied.

Results

A total of 101 patients underwent IPAA. Early complications (≤30 days) occurred in 72 (71.3%) patients, mostly Clavien-Dindo grades 1 and 2. No significant risk factors were associated with early complications. Among the late complications, pouchitis was the most common complication (n = 37, 36.6%), followed by anastomotic stricture (n = 27, 26.7%). Pouch failure was seen in 11 (10.9%) patients. No significant factors were found to be associated with the development of pouchitis. Pelvic sepsis (odds ratio (OR) = 2.704, 95% confidence interval (CI) = 1.041-7.022, p = 0.041) and handsewn anastomosis (OR = 3.943, 95% CI = 1.093-14.229, p = 0.036) were significantly related to the development of anastomotic stricture and pouch-vaginal fistulae, respectively.

Conclusions

The most common early and late complications following IPAA were pelvic sepsis and pouchitis, respectively. These complications were managed successfully with an acceptable pouch failure rate. No predictive factor was found to be significant with early complications. However, pelvic sepsis and hand-sewn anastomosis were associated with stricture formation and pouch vaginal fistulae, respectively.

## Introduction

The prevalence of ulcerative colitis (UC) is higher in Western countries compared to India. Changing dietary habits and lifestyles are projected to make India one of the countries with the highest disease burden of UC in the world [1,2]. About 20% to 30% of patients with UC require surgery at some point [3]. Restorative proctocolectomy with ileal pouch-anal anastomosis (IPAA) is the preferred surgical treatment for UC, offering disease-free bowel function, reducing the cancer risk without a permanent stoma, and lowering long-term costs. Nevertheless, IPAA is not free from complications that affect long-term outcomes. Reported early complications are pelvic sepsis, bleeding, and surgical site infection, among others. The long-term complications include pouchitis, pouch-anal anastomosis stricture, pouch vaginal fistulae, and fecal incontinence. Many studies have reported acceptable complication rates, good functional outcomes, and good quality of life (QoL) after IPAA for UC [4,5]. Most of these studies are from Western countries. There are only a few studies from India on the outcomes of IPAA in UC patients [6,7]. This study was conducted at a tertiary care referral center in north India to identify the early and late postoperative complications of IPAA for UC and the associated predictors.

## Materials and methods

Study cohort

All patients with UC who underwent IPAA in the Department of Surgical Gastroenterology at Sanjay Gandhi Postgraduate Institute of Medical Sciences between 1995 and 2018 were included in this study. Ethical approval for this study was obtained from the Institutional Bioethics Committee (approval number: IEC-2024-64-MCh-EXP-57). Information about demographics, clinical parameters, operative details, and early and late postoperative complications were retrieved from a prospectively maintained hospital-based electronic database. Patients undergoing IPAA with diagnoses other than UC, Crohn’s disease, indeterminate colitis, or familial adenomatous polyposis were excluded. The extent of the disease was classified as ulcerative proctitis (E1), left-sided UC (E2), or pancolitis (E3) according to the Montreal Classification [8]. The indications were recorded as elective indications, which comprised failed medical therapy (steroid-refractory UC, steroid-dependent UC), dysplasia, and malignancy. Emergent indications were acute complications such as toxic megacolon, perforation, and acute severe ulcerative colitis (ASUC). ASUC was defined using the Truelove and Wits criteria [9]. The early complications were defined as complications within 30 days following IPAA. Early complications were graded according to the Clavien-Dindo classification [10]. Pelvic sepsis was defined as “Any infective process in the peri pouch area, detected during the investigation of clinical symptoms. This includes all abscess formations associated with or without anastomotic leak or purulent drain output.” Late complications were those that occurred during the follow-up period before or after stoma closure. Late complications included small bowel obstruction, pouchitis (diagnosed endoscopically and/or by histopathology), abscess, anastomotic stricture, and pouch failure. The anastomotic stricture was defined as a clinically significant stricture that requires endoscopic or surgical dilation. Pouch failure was defined as the need to construct a permanent ileostomy with or without excision of the ileal pouch [11]. A subjective scoring system on a scale from 0 to 10 was employed to evaluate patient satisfaction following surgery. Patients with a score of 9-10 were classified as very satisfied, those with a score of 6-8 as satisfied, and those with a score below or equal to 5 as poorly satisfied with surgery.

Surgical technique

All patients were referred to surgery by a medical gastroenterologist after initial medical management with steroids, immunosuppressants, and biologics. Most patients were tapered off steroids before pouch construction. The two-stage procedure was defined as a proctocolectomy with the creation of the IPAA and covering loop ileostomy as the first stage, followed by the ileostomy closure. The three-stage procedure involves a subtotal colectomy with end ileostomy and distal mucosal fistulae creation during the first stage, followed by restorative proctocolectomy with IPAA and covering loop ileostomy, and, finally, ileostomy closure. The IPAA was performed either by the stapled or handsewn technique. All IPAA procedures were performed using the open technique.

Statistical analysis

SPSS Statistics for Windows, Version 26.0 (IBM Corp., Armonk, NY, USA) was used for statistical analyses. Continuous variables are presented as mean and standard deviation (SD) or median (interquartile range (IQR)) depending upon normality status, while categorical variables are reported as raw values with percentages. Univariate analysis was performed using Pearson’s chi-square and Fisher’s exact tests. Odds ratios (ORs) and 95% confidence intervals (CIs) were calculated using univariate logistic regression analysis to assess the relationship between the outcome and potential influencing factors. Variables with a p-value <0.05 in the univariate analysis were selected for multivariate analysis. Statistical significance was set at p-values <0.05. Risk factors for early complications were analyzed using univariate analysis. Various late complications such as pouchitis, anastomotic stricture, and pouch vaginal fistulae were separately analyzed using univariate analysis.

## Results

A total of 101 patients underwent IPAA for UC. The median age of the patients was 32 years (range = 14-67 years; IQR = 26-44 years). The median preoperative disease duration was 48 months (range = 1.0-260.0 months; IQR = 23.3-118.8 months). The median follow-up period was 64 months (range = 6.0-273.0 months; IQR = 35.5-136.0 months). The median length of hospital stay after IPAA was 12 days (IQR = 9-18 days). The demographic and clinical characteristics of patients are described in Table 1.

**Table 1:** Demographic data and clinical characteristics (n=101). ^a^: Some patients had more than one comorbidity. UC = ulcerative colitis; ASUC = acute severe ulcerative colitis; IPAA = ileal pouch-anal anastomosis

Clinical characteristics	Number of patients, n (%)
Sex	
Male	69 (68.3)
Female	32 (31.7)
Age (median)	32 years (IQR = 26–44 years)
Comorbidities^a^	
Diabetes mellitus	5 (4.9)
Hypertension	3 (2.9)
Epilepsy	2 (1.9)
Bronchial asthma	2 (1.9)
Hyperthyroidism	1 (0.9)
Coronary artery disease	1 (0.9)
Time from diagnosis to surgery	48 months (IQR = 23.3–118.8 months)
Extraintestinal manifestations	16 (15.8 )
Blood transfusion before surgery	27 (26.7)
Extent of colitis	
E2	48 (47.5)
E3	53 (52.4)
Indications of surgery	
Steroid-dependent UC	48 (47.5)
Steroid-refractory UC	34 (33.6)
ASUC with perforation	5 (4.95)
Toxic megacolon	6 (5.9)
ASUC with bleeding	6 (5.9)
Associated colon carcinoma	1 (0.9)
Colonoscopy perforation	1 (0.9)
Surgery type	
Emergent	18 (17.8)
Elective	84 (83.1)

Indication of surgery and surgical procedure

**Figure 1:**
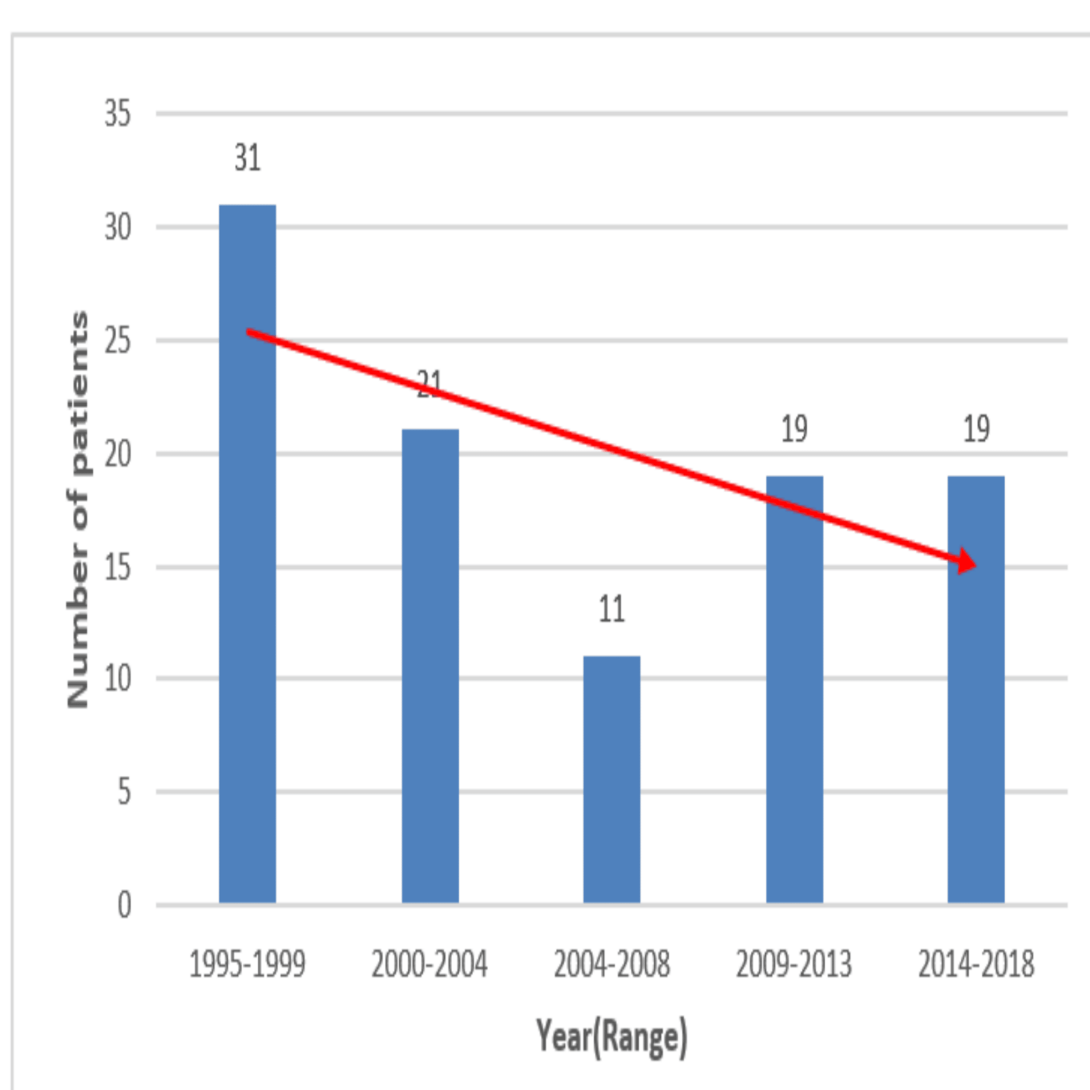
Bar chart showing the trend of Ileal pouch-anal anastomosis over time.

The indications and types of surgery are described in Table 1. A three-staged procedure was performed in 57 (56.4%) patients, while a two-staged procedure was performed in 44 (43.6%) patients. Most patients who underwent the three-stage procedure were steroid-refractory (n = 29, 50.8%) or had acute emergencies (n = 15, 26.3%). The two-stage procedure was primarily performed for steroid-dependent UC (n = 30, 68.1%). J-pouch was formed in 91 (90.1%)patients, while a W-pouch was formed in 10 (9.9%) patients. We performed a subgroup analysis to examine the complications rate between the J-pouch and W-pouch and could not find any significant difference. Most patients underwent IPAA using a double stapling technique (n = 74, 73.2%), while hand-sewn anastomosis was performed in 27 (26.7%) patients. A diverting loop ileostomy was performed for all patients following pouch-anal anastomosis. Three patients underwent a laparoscopic first-stage procedure (subtotal colectomy). The analysis revealed that in the initial five years, the number of pouches was high and decreased afterward, but it remained in a similar range from 2009 onward. The year-wise trend is shown in Figure 1.

Early postoperative complications after IPAA

In total, 72 (71.3%) patients experienced early postoperative complications (Table 2). The majority had Clavien-Dindo grade 1 (n = 27, 26.7%) and grade 2 morbidities (n = 24, 23.7%). Grade 3 and 4 morbidities were encountered in 17 (16.8%) and 3 (2.9%) patients, respectively. The most common complication was pelvic sepsis, observed in 26 (25.7%) patients. Of the 26 patients with pelvic sepsis, 15 had collections in the peri-pouch area, five had demonstrable pouch leaks, and six patients had purulent drain output. Of the 26 patients, seven required percutaneous drainage of collections, three required re-exploration, and the remainder were managed conservatively. Unfortunately, one patient died after IPAA in the immediate postoperative period due to anastomosis leak and sepsis. Overall, 10 (9.9%) patients required re-exploration in the early postoperative period for missed enterotomy (five cases), pelvic sepsis (three cases), and bleeding (two cases). Various risk factors analyzed to see their association with early complications are shown in Table 3. None of the factors were significantly associated with early complications on univariate analysis.

**Table 2:** Early postoperative complications (≤30 days). ^a^: Some patients had more than one complication.

Complications^a^	Number of patients, n (%)
Pelvic sepsis	26 (25.7)
Surgical site infection	21 (20.7)
Small bowel obstruction	12 (11.8)
Hemorrhage	10 (9.9)
From pouch (staple line)	4 (3.9)
Mesentery	2 (1.9)
Source not identified	4 (3.9)
Pneumonia	2 (1.9)
Missed enterotomy	5 (4.9)
Seizure	1 (0.9)

**Table 3:** Risk factors for early complications: factors and their significance (univariate analysis). Values are presented as odds ratio (95% confidence interval). IPAA = ileal pouch-anal anastomosis; CMV = cytomegalovirus; E2 = left-sided ulcerative colitis; E3 = extensive ulcerative colitis; UC = ulcerative colitis; TNF = tumor necrosis factor

Variable	Univariate analysis	P-value
Male	1.484 (0.600-3.672)	0.392
Age (≥35 vs. <35)	1.453 (0.601-3.523)	0.406
Albumin (<3.5 vs. ≥3.5 )	1.578 (0.549-4.540)	0.342
Systemic steroid within 1 month before IPAA	2.139 (0.841-5.550)	0.113
Anti-TNF therapy within 1 month before IPAA	0.510 (0.107-2.435)	0.391
Types of pouches		
J-pouch	2.792 (0.742-10.496)	0.117
W-pouch	0.358 (0.095-1.347)	
Extent of colitis		
E2	0.462 (0.191-1.118)	0.074
E3	2.291 (0.946-5.547)	
Indications of surgery		
Elective	0.863 (0.280-2.662)	0.961
Emergent	1.058 (0.340-3.292)	
Extraintestinal manifestation	1.129 (0.430-2.963)	0.807
Surgical procedure		
Two stage	0.629 (0.264-1.198)	0.294
Three stage	1.598 (0.667-3.782)	
Hand-sewn anastomosis	0.741 (0.286-1.916)	0.535

Late postoperative complications after IPAA

Late complications were observed in 59 (58.4%) patients. The late complications were as follows: pouchitis (n = 37, 36.6%), anastomotic stricture (n = 27, 26.7%), pouch-vaginal fistula (n = 11, 10.9%), pouch-perianal fistulae (n = 3, 2.9%), small bowel obstruction requiring re-exploration (n = 17, 16.83%), incisional hernia (n = 6, 5.9%), impotence (n = 1, 0.9%), and urinary retention requiring clean intermittent self-catheterization (n = 2, 1.9%). We analyzed various long-term complications separately, as outlined below.

Pouchitis

Table 4 highlights the risk factors analyzed to assess their association with pouchitis. On univariate analysis, none were found to be significant in our study. Of the 37 patients with pouchitis, 34 responded to conservative management, such as oral antibiotics, hydrocortisone enema, and mesalamine suppositories. Three patients required pouch excision.

**Table 4:** Factors analyzed for late complications (pouchitis, anastomotic stricture, and pouch-vaginal fistulae) and their significance (univariate analysis). Values are presented as odds ratio (95% confidence interval). ^a^: P-values <0.05. CMV = cytomegalovirus; E2 = left-sided ulcerative colitis; E3 = pancolitis

Variable	Univariate analysis		
	Pouchitis	Anastomotic strictures	Pouch-vaginal fistulae
Age (≥35 vs. <35)	1.093 (0.484-2.465)	1.219 (0.504-2.950)	2.395 (0.654-8.767)
Male gender	1.154 (0.480-2.775)	0.877 (0.336-2.288)	-
Extraintestinal manifestation	1.045 (0.346-3.153)	1.829 (0.593-5.637)	1.206 (0.235-6.187)
Blood transfusion	0.998 (0.400-2.490)	0.516 (0.199-1.336)	1.179 (0.282-4.929)
History of CMV infection	0.842 (0.221-3.207)	1.913 (0.495-7.389)	2.222 (0.408-12.113)
Indication of surgery			
Elective indication	1.329 (0.481-3.675)	0.553 (0.192-1.596)	0.954 (0.189-4.825)
Emergent indication	0671 (0.239-1.887)	0.499 (0.171-1.459)	0.973 (0.192-4.940)
Extent of involvement			
E3	1.105 (0.491-2.488)	0.789 (0.327-1.908)	0.596 (0.163-2.184)
E2	0.905 (0.402-2.037)	1.267 (0.524-3.062)	1.674 (0.458-6.118)
Types of pouch			
J	5.891 (0.715-48.506)	0.836 (0.200-3.495)	1.111 (0.127-9.711)
W	0.170 (0.021-1.398)	1.196 (0.286-5.003)	0.900 (0.102-7.836)
Anastomosis			
Stapled	0.788 (0.319-1.946)	0.820 (0.309-2.179)	0.254 (0.070-0.915)
Hand-sewn	1.269 (0.514-3.134)	1.219 (0.459-3.238)	3.943 (1.093-14.229)^a^
Stage			
Two stage	0.480 (0.206-1.117)	0.557 (0.222-1.400)	0.609 (0.173-2.143)
Three stage	2.083 (0.895-4.850)	1.795 (0.714-4.509)	1.642 (0.467-5.480)
Pelvic sepsis	1.714 (0.691-4.252)	2.704 (1.041-7.022)^a^	1.766 (0.472-6.606)

Pouch-Anal Anastomotic Stricture

Of the 27 patients with anastomotic stricture, 19 (70.4%) had stapled anastomosis, and eight (29.6%) had hand-sewn anastomosis. The various risk factors analyzed to assess their association with anastomotic stricture are depicted in Table 4. The presence of pelvic sepsis was significantly associated with the development of anastomotic stricture (OR = 2.704, 95% CI = 1.041-7.022, p = 0.041). All patients were subjected to a graded dilatation program. Overall, 24 (88.8%) patients underwent successful dilatation by endoscopic or surgical methods. The average number of dilation sessions in the present study was four. Three patients had unsuccessful dilatation; one of them required pouch excision due to severe pouchitis and coexisting pouch-vaginal fistulae. Two other patients were still living with a stoma at the time this study was completed.

Pouch-Vaginal Fistulae

In total, 11 (10.9%) patients developed a pouch-vaginal fistula during the follow-up. While in five patients it was reported before the stoma closure, six patients found it after stoma closure. The various risk factors analyzed to assess their association with pouch-vaginal fistula are depicted in Table 4. Hand-sewn anastomosis was the only factor significantly associated with the development of pouch-vaginal fistula in univariate analysis (OR = 3.943, 95% CI = 1.093-14.229, p = 0.036). Four of these patients required surgical repair, while two patients were managed with diversion ileostomy followed by restoration of continuity. One patient later had recurrent pouch-vaginal fistulae with severe pouchitis, leading to pouch excision. Two patients are still living with a stoma.

Pouch Failure

In the long term, 11 (10.9%) patients suffered from pouch failure. Various causes of pouch failure included pouchitis (three patients), pouch-vaginal fistulae (three patients), pouch peri-anal fistulae (two patients), anastomotic stricture (two patients), and fecal incontinence (one patient). Three patients had their pouches excised with end ileostomy. In one patient, the pouch-anal anastomosis was dismantled, and the end of the pouch was made into a stoma. In seven patients, the stoma could not be reversed.

Long-term outcomes

Follow-up data were collected from outpatient visit records, personal interviews, and telephone conversations. On analyzing the long-term outcomes, the average stool frequency was 6.18 ± 1.87/day. Daytime frequency was 4.05 ± 1.27/day, and nighttime stool frequency was 2.05 ± 2/day. Of the study population, patient satisfaction could only be assessed in 65 (64.3%) patients, who were contacted either in the outpatient department or via direct telephone conversation. Using a subjective scoring system, they were asked to rate their satisfaction with the surgery after IPAA on a scale of 0-10. Overall, 49 (75.3%) patients were either very satisfied or satisfied with the IPAA. Common reasons for dissatisfaction in the remaining patients were recurrent abdominal pain, increased stool frequency, and nocturnal incontinence.

## Discussion

Despite the emergence of biologics and improved medical management, IPAA remains the surgical treatment of choice for UC when surgery is warranted. IPAA offers a one-time solution for UC, potentially providing significant relief for this vulnerable patient population. The demographic parameters in our study were similar to those reported in other studies. The median age at the time of surgery was 32 years, similar to Pal et al. from India [12]. Of the study population, 16 (15.8%) patients had extraintestinal manifestations, but none presented with primary sclerosing cholangitis (PSC). Previous studies from Western countries have demonstrated that the prevalence of UC with PSC ranged from 13.3% to 21.8%, whereas it is uncommon in India [11]. Studies by Kedia et al. [1] and Singh et al. [13] from India reported lower frequencies of PSC at 0.40% and 0.39%, respectively.

In the present study, medically refractory UC was the most common indication for surgery, accounting for 81.1% of cases. Similarly, in studies by Lim et al. [14] and Zittan et al. [15], medically refractory UC was also the primary indication for surgery, comprising 83% and 74.9% of study populations, respectively. The initial first five-year higher number of patients reflects increased referral and non-availability of medical treatment for better control and remission such as biologicals, antimetabolites, and immunosuppressants. In our study, the majority of patients underwent three-staged procedures. In the study conducted by Zittan et al. [15], 37.6% of patients underwent three-stage procedure, 31.3% underwent a modified two-stage procedure, and 29.4% underwent two-stage procedure. The J-pouch was preferred in our study, although 10 patients had a W-pouch performed in the first few years. The J-pouch is the preferred type of reconstruction in other studies as well, as it is technically easier and has widely acceptable functional outcomes. In their meta-analysis, Lovegrove et al. [16] demonstrated similar outcomes for all three types of pouches. In the present study, 74 (73.2%) patients underwent stapled anastomosis, while the remainder underwent hand-sewn anastomosis. Similarly, in a study conducted at the Cleveland Clinic, 87% of patients received stapled anastomosis, while 13% underwent hand-sewn anastomosis [11]. In this study, there were no significant differences in long-term outcomes between stapled and hand-sewn anastomosis, except for pouch-vaginal fistula, which was more commonly associated with hand-sewn anastomosis than with stapled anastomosis.

In the largest study (n = 2,959) by Fazio et al. [11], the mortality rate in the perioperative period (<30 days) was 0.1%. However, in the present study, it was 0.9%, which seems higher and may reflect the small sample size. In the present study, 72 (71.8%) patients experienced early morbidity. The early morbidity rate in various studies ranged from 20% to 50% (Table 5). Although we observed a higher rate of overall early complications, only 21% were Clavien-Dindo grades 3 and 4, while the remaining 51% were Clavien-Dindo grades 1 and 2 complications. McCombie et al. [17] observed grade 3-5 complications in 7.4% of patients. In the present study, a missed enterotomy was the most common cause of re-exploration (n = 5, 4.9%). In contrast, in the series by Kampka et al. [18], the most common reason for reoperation was an intra-abdominal/pelvic abscess (n = 6, 42.9%). Unlike in Western studies, the majority of patients with pelvic sepsis did not require re-exploration and were managed with percutaneous drainage.

**Table 5:** Published series on early complications after IPAA. IPAA = ileal pouch-anal anastomosis; SSI = surgical site infections; SBO = small bowel obstruction

Studies	Early complications							
	Sample size	Pelvic sepsis	SSI	Pneumonia	Bleeding	SBO	Re-exploration	Mortality
Fazio et al., 2013 [11]	2,959	329 (11%)	214 (7.2%)	-	106 (3.6%)	144 (4.9%)	-	4 (0.1%)
Lim et al., 2021 [14]	212	23 (10.8%)	20 (9.4%)	-	-	14 (6.6%)	-	-
McCombie et al., 2016 [17]	758	135 (17.8%)	108 (14.2%)	20 (2.7%)	-	134 (17.7%)	33 (4.4%)	-
Ryoo et al., 2014 [19]	121	9 (7.4%)	13 (10.7%)	-	9 (7.4%)	8 (6.6%)	-	1 (0.8%)
Present study, 2024	101	26 (25.7%)	21 (20.8%)	2 (2%)	10 (9.9%)	12 (11.8%)	10 (9.9%)	1 (0.9%)

Despite the increased morbidity, the mortality rate was low and comparable with the published series. Our study did not find any significant factors associated with developing early complications. In contrast, various studies have reported that older age and preoperative steroid use are linked to the development of early complications [14,15,17,19]. An increased rate of early complications in our study can be multifactorial. Although all patients in our study were tapered off steroids before pouch construction, many studies indicate that steroids are a significant risk factor for early complications following IPAA. Another factor contributing to the increased rate of early complications in our study was the patients’ general status. Although serum albumin levels were optimized before pouch construction, other factors, such as sarcopenia and American Society of Anesthesiologists grade, were not considered during the analysis. Many of our patients had compromised nutritional reserves that were not accurately reflected by serum albumin values. Additionally, although most patients underwent elective procedures, many can be accurately considered semi-elective procedures.

Late complications were observed in 59 (58.4%) patients. Comparisons of late complications observed in different studies are outlined in Table 6. On long-term follow-up, pouchitis was encountered in 37 (36.6%) patients. This rate is consistent with findings reported in other studies [20,21]. Several studies have identified various factors linked to the development of pouchitis, such as extraintestinal manifestations, pancolitis, a history of PSC, and prior treatment with anti-tumor necrosis factor before colectomy [21-23]. In the present study, we found no significant risk factors associated with pouchitis on univariate analysis. Only three (2.97%) patients required pouch excision due to severe pouchitis and associated fistulae, similar to the findings published in a previous study [11].

**Table 6:** Published series on late complications after IPAA. IPAA = ileal pouch-anal anastomosis; SBO = small bowel obstruction

Studies		Late complications					
	Sample size	Pouchitis	Anastomotic stricture	Pouch-vaginal fistula	Pouch anal fistula	Pouch failure	SBO
Fazio et al., 2013 [11]	2,959	1,063 (35.9%)	331 (11.2%)	-	37 (1.3%)	151 (5.1%)	144 (4.9%)
Lim et al., 2021 [14]	212	83 (39.2%)	33 (15.6%)	-	26 (12.3%)	9 (4.2%)	29 (13.7%)
McCombie et al., 2016 [17]	121	60 (49.6%)	17 (14%)	-	30 (24.8%)	14 (11.6%)	42 (34.7%)
Ryoo et al., 2014 [19]	71	17 (23.9%)	2 (2.8%)	5 (7%)	4 (5.6%)	02 (2.8%)	10 (14.1%)
Present study, 2024	101	37 (36.6%)	27 (26.7%)	11 (10.9%)	3 (2.9%)	11 (10.9%)	17 (16.8%)

The rate of anastomotic stricture reported in various studies ranges from 2% to 38% [24]. In the present study, 27 (26.73%) patients developed anastomotic stricture. In our research, pelvic sepsis was significantly associated with anastomotic stricture, consistent with the findings of Lewis et al. [25]. Other studies have demonstrated an association between female gender, pelvic sepsis, and hand-sewn anastomosis with the development of anastomotic stricture [26,27]. Most of our patients responded to endoscopic or surgical dilation, with only three patients experiencing treatment failure. Similar results were observed in other studies, demonstrating a good response of anastomotic strictures to dilation [19]. Our results indicate that most anastomotic strictures, which are typically non-fibrotic, can be successfully treated with dilation programs.

The overall risk of pouch-vaginal fistula following IPAA ranges from 4% to 16%. Pouch failure is observed in 21% to 30% of patients with pouch-vaginal fistula [28,29]. It is challenging to manage and is an essential factor leading to pouch failure [29]. In the present study, 11 (10.8%) patients had pouch-vaginal fistulae. In the present study, hand-sewn anastomosis was found to be a risk factor for pouch-vaginal fistula although the CI was wide. Mallick et al. [28] found that pouch-vaginal fistulas are also most common in patients with a history of pelvic sepsis. In the present study, 9 out of 11 patients (75%) with pouch-vaginal fistulas required some form of intervention, either repair or stoma creation, similar to findings reported in the literature [28].

While multivariate analysis can offer valuable insights into the relationships between multiple variables for the analysis of various early and late complications, we decided against using it in our study. This is due to the limited sample size and the lack of significant factors identified in the univariate analysis.

The present study showed pouch failure in 11 (10.9%) patients. The incidence of pouch failure was reported as 5.1% by the Cleveland Clinic, 7.7% by the UK Pouch Study Group, and 5.9% by Chapman et al. [30,31]. Heuthorst et al. [32] showed that pouch failure was significantly correlated with fistulae and pelvic sepsis. Similar results were also observed in our study, with all patients with pouch failure having pelvic sepsis in the immediate postoperative period, and 6 out of 11 patients had pouch-related fistulae. The average stool frequency was 6.18 ± 1.87/day, Daytime frequency was 4.05 ± 1.27/day, and nighttime stool frequency was 2.05 ± 2/day. In a study conducted at Cleveland Clinic, average daytime and nighttime frequencies were 5.06 ± 1.8 and 1.36 ± 1.3, similar to our results [4]. In a study conducted in India by Rao et al. [6], the mean stool frequency was 7.2 stools per 24 hours. In Another study by Raviram et al. [7] from India, the median stool frequency was 7 per 24 hours. Due to the study’s retrospective design, we devised a subjective scoring system ranging from 0 to 10 to assess satisfaction after IPAA. In our study, 75% of patients were satisfied or very satisfied after surgery. Similarly, in a study by Rao et al. [6], 85% of patients reported being very satisfied with the procedure. In another study conducted in India, Somashekar et al. [33] reported a mean stool frequency of 7, with 84% of patients expressing satisfaction with the surgery.

Our study has some limitations. First, its retrospective design results in bias and misclassification as many follow-up data were extracted from follow-up records. Second, the number of patients included in our study was smaller compared to the Western series. Third, the study period spanned over 30 years, during which changes in patient management, surgical techniques, and perioperative care could have influenced the results. Despite these limitations, this is the largest study conducted in India to date that describes various complications and analyzes risk factors associated with IPAA in patients with UC.

## Conclusions

In our study, both early and late complications were significantly high. The majority of early complications were minor except pelvic sepsis. Major late complications were pouchitis, strictures, pouch-vaginal fistulae, and pouch failure. No factors were found to be significantly associated with early complications. However, pelvic sepsis and hand-sewn anastomosis were significant predictive factors for the development of anastomotic stricture and pouch-vaginal fistulae, among late complications. By keeping the predictive factors in mind, surgeons may anticipate complications, which may help in performing prompt and judicious interventions to deal with these complications to improve the outcome. These factors may also help to take preventive measures during surgery to minimize morbidity. The result of our study will guide surgeons to be aware of possible complications of IPAA in UC and how to minimize and manage them.
